# Supplementary material for: Chromothripsis during telomere crisis is independent of NHEJ, and consistent with a replicative origin
Source: Genome Res. 2019 May;29(5):737–49. doi: 10.1101/gr.240705.118 (PMC6499312; doi:10.1101/gr.240705.118)
Supplement: Supplemental Material [file supp_gr.240705.118_Supplemental_file_1.zip › contigs/annotated_contigs/DB79/contig.3.DB79_length_290_mean_cov_5.75172413793.docx]

**DB79_length_290_mean_cov_5.75172413793**

```
CTCAGCTCACTGCAGCCTCTGCCTCCCAGGTTCAAGAGATTCTCCTGCCTCAGCCTCCAGAGTAGATGGGATTACAGGTGCCTGCCACC
 >chr22:18129551-18129722 + E=3e-80 p=0e+00
ACGCCCAGCTAATTTGTGTGTGTGTGTGTGTGTGTGTGTGTGTGTATAAACTACATATATATATATATATATA|TATAT|ATATATATA
                                                                          >chr1:7435382
TATATATATATTTTTTTTTTTTTTTTTTTAAAAAT|TGGTTCTCTCTG|TGTTTCCCAGGCTGGTGTGCAGTGGGGGAATACTGGCTCA
5-74353874 - E=4e-15                            >chr6:108301144-108301188 + E=4e-06
CTGC|TAGTGAGTAGCGGGGGCTGCAGG
```
